# Biometrics and Policing: A Protocol for Multichannel Sensor Data Collection and Exploratory Analysis of Contextualized Psychophysiological Response During Law Enforcement Operations

**DOI:** 10.2196/resprot.7499

**Published:** 2017-03-17

**Authors:** Robert D Furberg, Travis Taniguchi, Brian Aagaard, Alexa M Ortiz, Meghan Hegarty-Craver, Kristin H Gilchrist, Ty A Ridenour

**Affiliations:** ^1^ Digital Health & Clinical Informatics RTI International Research Triangle Park, NC United States; ^2^ Policing Research Program RTI International Research Triangle Park, NC United States; ^3^ Engineered Materials, Devices and Systems RTI International Research Triangle Park, NC United States; ^4^ Behavioral & Urban Health Program RTI International Research Triangle Park, NC United States

**Keywords:** psychophysiology, law enforcement, sensor, wearable, clinical trial, digital health

## Abstract

**Background:**

Stress experienced by law enforcement officers is often extreme and is in many ways unique among professions. Although past research on officer stress is informative, it is limited, and most studies measure stress using self-report questionnaires or observational studies that have limited generalizability. We know of no research studies that have attempted to track direct physiological stress responses in high fidelity, especially within an operational police setting. The outcome of this project will have an impact on both practitioners and policing researchers. To do so, we will establish a capacity to obtain complex, multisensor data; process complex datasets; and establish the methods needed to conduct idiopathic clinical trials on behavioral interventions in similar contexts.

**Objective:**

The objective of this pilot study is to demonstrate the practicality and utility of wrist-worn biometric sensor-based research in a law enforcement agency.

**Methods:**

We will use nonprobability convenience-based sampling to recruit 2-3 participants from the police department in Durham, North Carolina, USA.

**Results:**

Data collection was conducted in 2016. We will analyze data in early 2017 and disseminate our results via peer reviewed publications in late 2017.

**Conclusions:**

We developed the Biometrics & Policing Demonstration project to provide a proof of concept on collecting biometric data in a law enforcement setting. This effort will enable us to (1) address the regulatory approvals needed to collect data, including human participant considerations, (2) demonstrate the ability to use biometric tracking technology in a policing setting, (3) link biometric data to law enforcement data, and (4) explore project results for law enforcement policy and training.

## Introduction

Stress experienced by law enforcement officers is often extreme and is in many ways unique among professions. Unlike in most jobs, police officers are regularly exposed to violence, human suffering, and death, and they routinely have to deal with unpredictable and uncontrollable events [1,2]. In addition, many officers are required to work nonstandard work schedules (i.e., rotating or irregular, evening, night, or split shifts), for which the negative effects on individuals and families are well documented [3-6]. Research has found that unpleasant interactions with the public and exposure to disturbing situations (e.g., responding to incidents involving child victims) are important sources of stress among police officers [1]. In turn, high levels of work-related stress can lead to negative health outcomes for officers, including fatigue, insomnia, depression and anxiety, and a range of psychosomatic issues such as lower back pain, headaches, and digestive problems [7-9]. Stress also affects health through its relationship to harmful behaviors, such as increased alcohol use, smoking, and lack of physical leisure activities [9], as well as eating a higher-fat diet [10]. In the longer term, chronic work stress can increase the risk of heart disease and diabetes [11].

Work stress affects not only officers’ health, but also their job performance, which can influence their interactions with community members and ultimately the safety of the communities they serve. Some laboratory-based studies have found a curvilinear relationship between stress and performance; however, in the field, especially when complex tasks are at hand, moderate to high levels of stress have been found to be detrimental to performance [12]. For instance, job-related stress is positively associated with the likelihood of being injured on the job [13], absenteeism [14], and turnover intentions and behaviors [15]. Work-related stressors can also correspond with negative emotions, such as aggression and hostility, increasing the likelihood of interpersonal problems with coworkers [16]. Stress can also affect interactions with the community; for instance, officers who report more burnout—the result of prolonged exposure to work stress—report more favorable attitudes toward the use of violence [17].

Critical for our study, a significant body of research indicates that exposure to stress can directly or indirectly affect decision-making capabilities. When exposed to a threatening or stressful event, the sympathetic nervous system is activated, while the parasympathetic nervous system (responsible for calming the body) is deactivated [18]. Greater perceptions of threat correspond to higher levels of sympathetic nervous system arousal (also known as fight-or-flight responses) and the release of stimulant hormones (e.g., cortisol, adrenaline) that can impair fine motor skills, reduce focus, and negatively affect decision making [19,20]. Specifically, the release of adrenal stress hormones constricts blood vessels and decreases oxygen levels in the prefrontal cortex, which limits the individual’s ability to access stored memories and experiences [21].

Moreover, heightened physiological arousal may drive individuals into a state of hypervigilance, in which they rely more on their reactive limbic system than on their frontal lobe, which is used for reasoning and analytical thinking [22]. Baradell and Klein [23] argued that stressful events encourage “resource depletion,” in which cognitive resources are dedicated to managing the anxiety that is generated from exposure to a stressful event. Resource depletion interferes with the capacity to consider options or alternatives or to scan the environment for additional information when interpreting a situation. Fight-or-flight responses have also been linked to perceptual distortions (e.g., tunnel vision, narrowing of auditory information) [24,25], which can slow reaction time and impede the ability to identify and understand potentially life-threatening cues. Evidence also suggests that stress negatively affects sleep quality [26], which in turn degrades individuals’ abilities to perform well on complex decision-related tasks [27].

Recent high-profile events involving law enforcement’s deadly use of force have intensified scrutiny on American policing and initiated national conversations about the need for systematic police reform. Larger discourse related to the “crossroads in American policing” has criticized law enforcement for what is perceived to be poor decision making among officers during several highly publicized events, including the deaths of Eric Garner and Michael Brown [28]. These perceptions have led some practitioners and social commentators to campaign for improved decision-based training or other efforts to enhance decision-making skills among officers [29]. As we have described, stress is a key component of poor decision making. Fortunately, there is an extensive knowledge base pertaining to ways that organizations can ameliorate the effects of stress on employees, and work is being done to translate this literature into a police organizational setting.

### Organizational Responses to Stress

Robson and Manacapilli [30] described several ways in which employees within an organization become competent in stress management skills: (1) prehire screening designed to select individuals more likely to succeed in stressful environments, (2) deliberate removal of those who are not operating well under stress, (3) allowing employees who are unable to cope with stressors to select themselves out of the job, and (4) using training to minimize the impacts of stress.

Law enforcement agencies engage in a battery of prehire tests, some of which are relevant to screening out individuals who do not possess the disposition necessary to cope with the stresses of policing. Options 2 and 3 can be more challenging. These options can be expensive (e.g., employee turnover), difficult to implement (e.g., removing an officer with civil service protection), or outside of the agency’s control (e.g., officer volunteering to leave). From a pragmatic perspective, developing behavioral and cognitive skills to minimize the damages of stress is the most efficient method of dealing with officer stress. This takes advantage of the human capital contained within well-trained officers, leverages the training capabilities of modern law enforcement agencies, and makes use of a growing body of evidence-based trainings.

In other settings, workplace-based stress management programs have been found to improve a variety of employee outcomes, including reduced stress and enhanced emotional and physical health (e.g., reduced blood pressure) [31]. There are multiple types of workplace health trainings, and each has been associated with different outcomes. For example, cognitive behavioral training is effective in improving perceived quality of work life, and psychological resources and responses [32]. Trainings designed to teach employees effective coping strategies, appropriate for different kinds of workplace stressors, reduce the effect of potential stressors on health and well-being.

Organizations can also undertake more substantial organizational changes to address known stressors. For example, shift work is a recognized contributor to employee stress and negative health outcomes [33]. Research on police shift work identified certain kinds of work schedules that minimize, to the extent possible, the negative effects of shift work and rotating schedules [34]. The extent to which these organizational changes are effective, however, is largely unknown. This demonstration project seeks to develop a methodology that could be used to evaluate interventions of these types.

### Study Objective

Recent advances in smartphone and wristband sensor technology allow for the study of affect from several important perspectives that will enable understanding of the interplay between affect and physical health in our everyday lives. First, advances in wearable sensor technology allow for the collection of precise and reliable data on psychophysiological indicators (e.g., electrodermal activity [EDA] and galvanic skin response, heart rate, heart rate variability, physical activity) that have been linked to affect in previous research [35-37]. These devices provide a continuous stream of biometric data and can be worn throughout the day with no interruption to daily routines. The study of affect in the naturalistic setting, outside of the laboratory, is particularly important, as little is known about the day-to-day physiology of affect, largely due to the low levels of precision and high levels of noise in data collected to date in environments where people are ambulatory.

This demonstration project is based on 2 established research findings: (1) stress has negative implications for both individuals and the organization they work in, and (2) stress management training has shown positive results for managing stress and ameliorating the negative impact of stress. Broadly, our research objective for this study is to demonstrate the practicality and utility of biometric sensor-based research in a law enforcement agency. Although past research on officer stress is informative, it is limited. Most studies have measured stress using self-report questionnaires or were observational studies with limited generalizability. Objective physiological assessment of individuals in occupational settings other than law enforcement has demonstrated the utility of heart rate variability analysis to identify effort at work [38], psychological distress [39], and self-reported burnout [40]. We know of only 2 other research studies that have attempted to track direct physiological stress responses within an operational police setting [41,42].

Moreover, previous studies on law enforcement stress management were hampered by poor design and evaluations with low statistical power. The outcome of this project will have an impact on both practitioners and policing researchers. To do so, we will establish a capacity to conduct this type of research by obtaining complex, multisensor data; processing complex datasets; and establishing the methods needed to conduct idiopathic clinical trials on behavioral interventions in similar contexts. Our research objectives are to (1) explore the viability of using commercially available biometric tracking technology in a law enforcement setting, (2) determine whether biometric data can be linked to law enforcement data stored in a records management systems, (3) explore the results in the context of policy and training implications for law enforcement agencies, and (4) document the viability of using biometric-related data in an evaluation framework in a law enforcement agency.

The study will use an innovative and methodologically sophisticated research design to address a critical issue in contemporary law enforcement: officer stress. Previous research has shown that police officers are vulnerable to a variety of stressors that are a result of organizational issues, operational experiences, or individual-level characteristics. Likewise, an extensive body of research has demonstrated linkages between stress and several deleterious officer health-related outcomes such as fatigue, insomnia, and anxiety. Importantly, stress can also manifest itself as impaired decision making. Given the current national scrutiny on American policing and the perceived need for enhanced decision-based training, it is imperative that researchers strengthen the knowledge base about both officer stress and stress management techniques appropriate for police agencies.

## Methods

### Study Design

This pilot study is intended to explore the feasibility of using wrist-worn biometric sensors in law enforcement. We will use nonprobability convenience-based sampling to recruit 2-3 participants from the police department in Durham, North Carolina, USA.

### Recruitment

We will rely on agency executive staff to distribute recruitment material to all sworn patrol officers and to assist in identifying those who may be interested in participating. The recruitment email will include the study goals, expectations, and compensation. If the individual is interested, we will schedule an in-person or telephone meeting to discuss their participation further. To prevent any coercive effects, we will reach out directly to potential participants. During the meeting we will discuss the following: the study’s goals; that this is not a medical study; the potential risks to participants; expectations for wearing the device; compensation and compensation structure; the number of contacts needed during the study; other data we will collect from the agency; and our inability to provide true confidentiality. At this meeting, we will also demonstrate use of the device to the participant.

### Inclusion and Exclusion Criteria

Recruitment will be limited to sworn officers, employed by, and in good standing with, the Durham Police Department in Durham, North Carolina. Among sworn officers, we will limit recruitment to those officers who operate in a standard patrol capacity.

### Ethics and Confidentiality

This study has been reviewed and approved by the RTI International institutional review board. Device handling and data management protocols were reviewed by RTI’s Cloud Computing Security Team.

### Data Collection

We will collect three major types of data (biometric, operational, and spatial) from a variety of sources for each officer over 4 weeks. We describe each data type and source in more detail below.

#### Biometric Data

The use of biometric data will provide a highly nuanced look at physiological stress response. The Empatica E4 (Empatica Inc, Boston, MA, USA) is a wrist-worn wireless device capable of monitoring physiological signals through multiple sensors. EDA is used to determine the occurrence of stress or excitement by monitoring involuntary changes in skin conductivity mediated by the wearer’s sympathetic nervous system. Heart rate and other cardiovascular measures are derived from the blood volume pulse signal, measured through a *photoplethysmographic* sensor. A 3-axis accelerometer records the user’s physical activity, and an infrared thermophile captures the user’s skin temperature. All recordings have a time stamp, which we will use as the merge key for the agency data described below. Earlier versions of the E4 have been used in prior research studies to measure stress, skin conductance, and heart rate [43,44].

When a person is exposed to a stressor, the autonomic nervous system is triggered, resulting in the secretion of hormones into the bloodstream. These hormones lead to increased blood pressure, increased muscle tension, and changes in heart rate and heart rate variability [38]. This process is commonly known as the fight-or-flight reaction. When the stressor is no longer present, a negative feedback system stops this response and reestablishes the typical physiological balance for the individual.

During the last few decades, researchers have used subtle changes in heart rate to measure mental stress. Heart rate variability is calculated based on variation of time, in milliseconds, between 2 heartbeats. This parameter provides an observation of the heart’s ability to respond to normal regulatory impulses and can reflect changes in stress while other physiological parameters remain in normal or accepted ranges.

EDA, or galvanic skin response, describes involuntary changes in the electrical properties of the skin. Increased EDA indicates sympathetic activation or arousal and is widely used as a sensitive index of emotional processing. EDA is considered the most useful index of changes in sympathetic arousal that are traceable to emotional and cognitive states and is the only autonomic psychophysiological variable that is not contaminated by parasympathetic activity.

In the study, we will give participating officers a copy of the E4 device manual and training to operate the device. Officers will be instructed to wear the device and initiate a recording session at the beginning of each 12-hour shift.

#### Operational Data

Like most modern police departments, the Durham Police Department uses computer-aided dispatch (CAD) to facilitate the management and safety of patrol officers. CAD data capture a wide array of information on calls received from the public, calls initiated by officers, and the status of agency resources. For the purposes of this study we will extract 4 pieces of information. First, we will obtain officer identifiers to limit data processing to only the study participants. Second, we will extract relevant call details such as the type of event and the priority with which it is dispatched. We hypothesize that calls with higher priority or calls that involve violence or a higher degree of situational uncertainly will be associated with greater physiological reaction. Third, we will capture information on participant status. CAD data allow us to know what each participant is doing at any given time. Officers can be assigned to a call (dispatched), en route to a call, at the scene of a call, transporting an arrestee, or cleared from a scene. We will broadly classify these as allocated (assigned to a call) or unallocated (unassigned to any specific activity). We will further refine this classification and investigate the relationship between calls that are self-dispatched (e.g., a traffic stop) or citizen initiated (e.g., a call of a fight in progress). Fourth, we will extract various time components such as when the call is received, when the participant is dispatched, and when the participant has returned to service. We will use this time to link biometric data with participant activity data.

#### Spatial Data

Related to the CAD system, automated vehicle-locating technology is also used by the Durham Police Department to aid in police operations, especially with regard to dispatch decision making. Global positioning system (GPS) devices located in the patrol vehicle routinely report on the vehicle’s location, direction, speed, and heading. These data are stored by the Durham Police Department for a period of 30 days. The Durham Police Department will export these data for us regularly during the study period so that we will have full coverage of all participants during the biometric monitoring phase. The automated vehicle-locating data extract comes as a comma-separated values (CSV) file, where each row represents one location ping. Ping frequency depends on the speed of the vehicle; location is updated more frequently when the vehicle is moving and less frequently when the vehicle has been stationary. We will extract 4 pieces of information from this dataset: (1) vehicle *xy*-coordinates for location awareness, (2) time of ping occurrence to allow syncing with biometric data, (3) status and call identifier to understand what participants were doing, and (4) officer identifier to extract data on participants.

**Table 1:** Multichannel data sources that will be assimilated in the Biometrics &amp; Policing Demonstration pilot.

Data	Source	File type	Resolution	Use
Blood volume pulse	Biometric sensor	CSV^a^	64 Hz	Cardiovascular function
Interbeat interval	Biometric sensor	CSV	N/A^b^	Heart rate variability
Physical activity and posture	Biometric sensor	CSV	32 Hz	Physical measure
Electrodermal activity	Biometric sensor	CSV	4 Hz	Stress metric
Skin temperature	Biometric sensor	CSV	4 Hz	Stress metric
Automatic vehicle locators	Agency	Spatial point	Approximately every 3-30 seconds	Correlation of location and stress
Calls-for-service	Agency	Spatial point	Event level	Workload, time on-call, time off-call, call ordering
Incident data	Agency	Spatial point	Event level	Area crime patterns and stress

^a^CSV: comma-separated values.

^b^N/A: not applicable.

### Study Outcomes and Data Analysis

#### Data Management and Analysis Plan

The proposed project will require the assimilation of the data sources shown in Table 1. This assimilation presents several data management challenges. We will use data from the Empatica E4 to capture and quantify the autonomic stress response an officer experiences during work activity. Data from the agency’s CAD system will determine the types and durations of the activities an officer performed during a shift. We will derive the approximate locations of participants using the automated vehicle-locating logs. This integration will allow us to characterize stress responses with relevant situational and locational context. Multimedia Appendix 1 shows the Biometrics & Policing Demonstration pilot project data model, detailing the concurrent acquisition of biometric, operational, and spatial data, and the processing pathways for each data type. Multimedia Appendix 2 shows how the processed data are fused for analysis and statistical modeling.

#### Signal Processing

The E4 provides EDA, skin temperature, and 3 axes of accelerometer data. We will use these raw data streams to compute parameters on a common time scale to facilitate statistical analysis. Nonoverlapping 20-second windows will be chosen. The skin temperature data will be mean averaged over this window. The 3 axes of accelerometer data will be condensed to a single activity parameter reflecting overall level of motion at the wrist. The EDA data cannot be condensed to a single average value, because the time-varying component of the EDA signal contains significant information. We will compute 12 different parameters from the raw EDA data stream. The raw EDA signal will be decomposed into 2 frequency bands: 0-0.04 Hz to capture the baseline value or general trend, and a 0.04-0.4 Hz band to capture the fluctuations in EDA around this baseline value. From the baseline signal, we will compute an average EDA baseline value over the 20-second time window, but this value is influenced by many confounding factors, such as the quality of the electrode contact to the wrist and skin moisture level. Therefore, this average baseline value may be normalized using both a difference and a *z* score based on the previous 20-minute time segment. The slope of the baseline EDA will also be computed on 2 different time windows to capture change in baseline EDA. The magnitude of the time-varying component of the EDA signal will be captured using the root mean square (RMS) value of the 0.04-0.4 Hz signal. This RMS EDA value will also be normalized using a difference and *z* score from the previous 20 minutes. Counting the number of peaks in the 20-second window exceeding specified thresholds captures the level of EDA fluctuation. We will report all of the computed metrics along with the corresponding time window. Events noted in the police log will be obtained on the same 20-second time scale.

### Detailed Description of Metrics

We will compute 19 metrics on 20-second nonoverlapping windows: *time* (3): time stamp, elapsed time, and elapsed time from midnight; *event* (1): event code; *bad data* (1): bad data value; *activity* (1): average activity count; *skin temperature* (1): average temperature; *EDA* (12): average EDA level, average EDA level difference, average EDA level *z* score, 20-second EDA level slope, 120-second EDA level slope, RMS EDA, RMS EDA difference, RMS EDA *z* score, number of peaks (high threshold), average peak height (high threshold), number of peaks (low threshold), and average peak height (low threshold).

#### Time

The start time of the Empatica data file will be taken as the start time of the derived metrics file. A *time stamp* will be computed every 20 seconds from that start time. Additionally, we will report both *elapsed time* and *elapsed time from midnight*.

#### Event

The *event code* for a 20-second window will be computed as the mode of the event assignment values (sampling rate=1 Hz). The event assignment values are as follows:

- 0.5: call was ultimately canceled
- 1.0: dispatch to arrive 1
- 2.0: arrive 1 to arrive 2, transport, *or* cleared (depends on what next event is)
- 2.5: arrive 2 to transport (if arrive 2 occurs before transport)
- 3.0: transport to cleared.

#### Bad Data

This data stream will be computed using an algorithm provided by Empatica that returns –1 if the data are bad and 1 if the data are good on 5-second intervals. The *bad data value* reported for a 20-second window will be the sum of the 4 values in the bad data stream spanned by this window:

- 4: all of the data are good (100% good)
- 2: one bad data point (75% good)
- 0: two bad data points (50% good)
–2: three bad data points (25% good)
–4: all of the data are bad (0% good)

#### Activity

The activity signal will be computed from the 3-axis accelerometer signal (sampling rate=32 Hz), where *activity* = √ *x*^2^– *y*^2^+ *z*^2^. The activity signal will be filtered to the 0.1-7 Hz band using a zero phase fifth-order Butterworth filter. Then, the activity count will be computed over 1-second nonoverlapping epochs as follows: *activity count* = ∑| *activity* |.

The *average activity count* will be the average activity count (sampling rate=1 Hz) over the 20-second window.

#### Skin Temperature

The *average temperature* will be the average temperature (sampling rate=4 Hz) over the 20-second window.

#### Electrodermal Activity

The EDA signal (sampling rate=4 Hz) will be filtered into 2 bands:

- *Baseline:* 100-point third-order polynomial filter (cutoff frequency=0.04 Hz); effectively, this signal will be the EDA signal that has been filtered to the 0-0.0 4 Hz band (i.e., the EDA level).
- *Bandpass:* baseline will be removed from the EDA signal, and the resulting signal will be further filtered using a 10-point third-order polynomial filter (cutoff frequency=0.4 Hz); effectively, this signal will be the EDA signal that has been filtered to the 0.04-0.4 Hz band.

The *average EDA level* will be the average of the baseline signal over the 20-second window. We will apply 2 forms of normalization to this metric to account for slow drift in baseline EDA value.

The *average EDA level difference* will be the current window minus the average of the windows from 20:20 to 0:20 previous.

The *average EDA level* z *score* will be the *z* score using the current window, where the population is considered to be the windows from 20:20 to 0:20 previous.

The *20-second EDA level slope* will be computed by fitting a line to the baseline signal over the 20-second window.

The *120-second EDA level slope* will be computed by fitting a line to the baseline signal over a window spanning 60 seconds before and after the current point.

The *RMS EDA* will be the RMS of the bandpass signal over the 20-second window. This reflects the amount of fluctuation in EDA.

The *RMS EDA difference* will be the current window minus the RMS of windows from 20:20 to 0:20 previous.

The *RMS EDA* z *score* will be the *z* score using the current window, where the population is considered to be windows from 20:20 to 0:20 previous.

The *number of peaks (high threshold)* will be the number of peaks in the 20-second window of the bandpass signal, separated by at least 1 second, that have a height ≥0.15 microsiemens. The *average peak height (high threshold)* will be the average of these peaks.

The *number of peaks (low threshold)* will be the number of peaks in the 20-second window of the bandpass signal, separated by at least 1 second, that have a height ≥0.02 microsiemens The *average peak height (low threshold)* will be the average of these peaks.

Multimedia Appendix 3 is a CSV file sample of the treated dataset. Variables have been cleaned, filtered, and conflated; this structure will be used as the basis for statistical analysis.

### Heart Rate and Heart Rate Variability Analysis

Before we can analyze heart rate variability, we will assess the quality of the *photoplethysmographic* sensor output, blood volume pulse, and interbeat interval. Session data for each participant will be visually inspected to provide a qualitative assessment of signal fidelity. We will then quantify 3 parameters of signal quality for the interbeat interval data: (1) the longest segment of valid data, (2) the mean and standard deviation of the gaps in the signal, and (3) the signal quality for each 20-second segment of interbeat interval data for consistency with the rating of the EDA signal. These qualitative and quantitative findings will inform the extent to which heart rate variability will be assessed and integrated with other measures.

### Data Fusion

Before within-participants analysis can be conducted, a variety of data sources must be combined. We will synchronize datasets to a common UNIX time stamp, align the sample rates, and produce a single, comprehensive artifact of filtered and preprocessed data for each officer’s shift suitable for statistical analysis.

### Mixed-Model Trajectory Analysis

Statistical analyses will be conducted separately per data stream as the outcome (e.g., average EDA level separately from activity). Mixed-model trajectory analysis (MMTA), derived from hierarchical linear modeling, will quantify each individual officer’s time series data at level 1, while aggregates of officer data across individuals are analyzed at level 2 [45-48]. Hence, observations are clustered within individuals. Maximum likelihood estimation and fit statistics test which model components provide (or do not provide) improved fit to the observed data. Compared with traditional hierarchical linear modeling, several adjustments are needed to counter the potential for biased estimates in small sample sizes [45-48]. First, during the model fitting process, the Kenward-Roger correction is used to reduce the probability of a type 1 error [49-51]. Then, after the best-fitting model is determined, restricted maximum likelihood estimation is used to derive parameter estimates, because the full maximum likelihood underestimates parameter variance due to how degrees of freedom are allocated and is especially problematic in small samples [52-54]. This modeling will facilitate understanding how factors such as (1) efficacy of intervention, (2) demographics (e.g., age), (3) officer activity (e.g., managing a domestic dispute vs patrolling a high-crime neighborhood), and (4) and error covariance structures (e.g., autoregressive integrated moving average vs Toeplitz) affect the officer’s physiological stress response. We will conduct analyses using SAS 9.3 (SAS Institute Inc).

Considerable evidence in using MMTA to quantify individual time series and outcomes is available from health and nonhealth fields (e.g., animal husbandry and genetics) in the context of best linear unbiased predictors [51,55,56]. Within-person MMTA can be represented by the single regression equation *Y*_it_ = *β*_0_ + *u*_0i_ + *β*_1_(time) + *u*_1i_(time) + *β*_2_ Intx_it_ + *β*_3_(Intx*time)_it_ + *e*_it_, where *Y*_it_ is an outcome for individual *i* at time *t*; the intercept for individual *i* is a function of the average sample intercept (*β*_0_) plus individual *i* ’s deviation from this average (*u*_0i_, which is assumed to have a normal distribution and each time point is uncorrelated with all others, using an error covariance structure to parse out autocorrelation); change in the outcome over time is a function of the sample average trend (*β*_1_) plus individual *i* ’s deviation from that trend (*u*_1i_, assumed to be normally distributed); differences between baseline and intervention phases are modeled as differences between phase intercepts (*β*_2_ Intx_it_) and trends (*β*_3_(Intx*time)_it_); and *e*_it_ denotes random error (an aggregate term that can be parsed into multiple sources of error). Important for analysis of biosensor data, “time” often fits the data best as a fixed effect per shift (i.e., time is coded as zero at the start of each shift) because of factors such as the accumulation of sweat at the sensors during a shift.

The term “mixed model” refers to categorization of model variables into fixed and random effects. Fixed effects involve variables assumed to have no measurement error, are constant across individuals, and have values that are equivalent across studies (e.g., most demographics, passage of time, study arm assignment). Random effects involve variables that represent random values from a larger population or involve generalizing inferences from the effect beyond the observed values (e.g., Gaussian psychological characteristics, an effect of time that varies across persons). While not discussed here due to space limits, this distinction is fundamental in terms of both analytic techniques and interpretation of results [57]. The planned analyses include statistical control variables (time; potential sources of stress that occur with every call to a scene, such as the initial communication and travel to the scene), while we will test highly stressful calls (e.g., a crime scene characterized by violence or high unpredictability) for the sources of stress and as fixed effects.

### Sample Size and Power

The stream of biosensor data will provide large statistical power to detect associations between physical stress response and specific experiences of police officers. The amount of biosensor data proposed to be collected will come from 12-hour shifts of 2-3 officers over 1 month (estimated to result in more than 500 hours of monitoring data). To illustrate this, we analyzed two brief splices of EDA data collected during the Biometrics & Policing Demonstration project.

Multimedia Appendix 4 shows the changes in the officer’s EDA throughout (A) a full shift (with 5 calls demarcated) and (B) a domestic dispute call versus (C) stolen vehicle recovery. MMTA results suggest a statistical model of the difference in EDA between a domestic dispute (B) and a less-stressful call (C). The respective mean EDA levels were 0.77 (SD 0.362) vs 0.11 (SD 0.095), reaching a *P*<.0001 level of statistical significance.

## Results

Data collection was conducted in 2016. We will analyze data in early 2017 and disseminate our results via peer reviewed publications in late 2017.

## Discussion

The Biometrics & Policing Demonstration project was developed to provide a proof of concept on collecting biometric data in a law enforcement setting. As part of this effort, we will recruit 3 officers from a large police department to participate in biometric data collection during their duty shifts over a 4-week period. This effort will enable us to (1) address the regulatory approvals needed to collect data, including human participant considerations, (2) demonstrate the ability to use biometric tracking technology in a policing setting, (3) link biometric data to law enforcement data, and (4) explore project results for law enforcement policy and training.

If this project is successful, future directions for expanding data collection will include obtaining regular self-report on affect and valence using ecological momentary assessment instruments; collecting biological specimens for assessment of concurrent biomarker variation alongside biometric response; applying instrumentation to individual participants with a GPS sensor to track the location of officers during time spent away from their patrol vehicle; incorporating nonworkday data collection to establish a nonoccupational baseline; and integrating biometric readings with body-worn or dash camera media.

Recommendations from the US President’s Task Force on 21st Century Policing [58] call for an enhanced focus on improving officer health and wellness. The proposed demonstration pilot will serve as a major step forward in understanding how to use an innovative but not field-tested data collection methodology using advanced biometric sensors. These efforts will establish a framework for other related lines of research. Programs designed to improve overall officer health, for example, can be better assessed using the nuanced information provided by biometric sensors. Officer-worn biometric sensors also have the long-term potential to directly enhance officer safety. At least one wearable biometrics company has explored the use of the devices for real-time monitoring of officer safety. The proposed study is a critical step in advancing our state of knowledge with regard to the utility of wearable biometrics in law enforcement and may lead to officer safety improvements.

Another relevant policy consideration is the potential for unmanaged stress to have negative implications on the operations of law enforcement agencies. High levels of stress can be associated with increased absenteeism, use of sick leave, and employee turnover. These employee actions can create management difficulties for agencies, which suggest that agencies have a vested interest in keeping employees healthy. Poor stress management for officers can also lead to acute problems, such as diminished decision-making capacity in highly charged situations, like use-of-force incidents.

Stress-compromised decision making can lead to negative police-community interactions. The relationship between high-stress situations and use of force is obvious. Officers may be more likely to use force inappropriately when operating under long-term stress. Nevertheless, use of force is relatively rare. A more frequent concern may be how stress affects the more routine interactions between officers and citizens. Officer stress may lead to police-community interactions with greater levels of tension and disrespect. These routine interactions have a profound effect on police-community relations. Efforts to better manage officer stress may ultimately improve police-community relationships.

## Appendix

Multimedia Appendix 1 Biometrics & Policing data model.

Multimedia Appendix 2 Combined variables inventory.

Multimedia Appendix 3 Sample of the treated data set.

Multimedia Appendix 4 Image showing electrodermal activity data from one officer's shift.

## Abbreviations

CAD: computer-aided dispatch
CSV: comma-separated values
EDA: electrodermal activity
GPS: global positioning system
MMTA: mixed-model trajectory analysis
RMS: root mean square
