# Supplementary material for: Biometrics and Policing: A Protocol for Multichannel Sensor Data Collection and Exploratory Analysis of Contextualized Psychophysiological Response During Law Enforcement Operations
Source: JMIR Res Protoc. 2017 Mar 17;6(3):e44. doi: 10.2196/resprot.7499 (PMC5375974; doi:10.2196/resprot.7499)
Supplement: Multimedia Appendix 1 [file resprot_v6i3e44_app1.pdf]

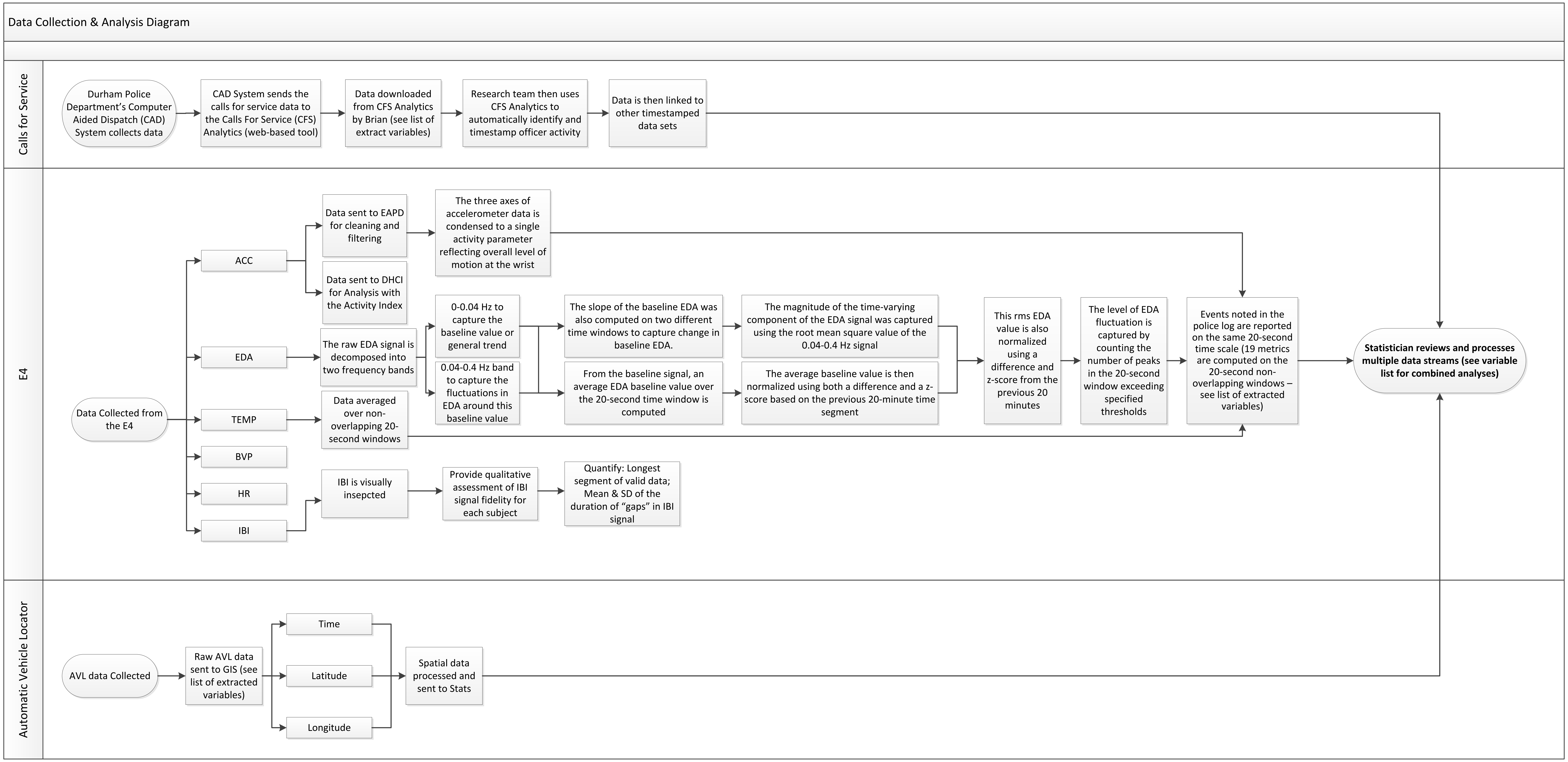

Multimedia Appendix 1. Data model illustrating the concurrent acquisition of biometric, operational, and spatial data, and the processing pathways for each data type for the Biometrics & Policing Demonstration pilot project.
