# Supplementary material for: Biometrics and Policing: A Protocol for Multichannel Sensor Data Collection and Exploratory Analysis of Contextualized Psychophysiological Response During Law Enforcement Operations
Source: JMIR Res Protoc. 2017 Mar 17;6(3):e44. doi: 10.2196/resprot.7499 (PMC5375974; doi:10.2196/resprot.7499)
Supplement: Multimedia Appendix 2 [file resprot_v6i3e44_app2.pdf]

Combined Data Analysis

| | |
|---|---|
| FullTimestamp | Timestamp (yyyy/mm/dd hh:mm:ss) |
| ElapsedSec | Elapsed time in sec |
| SecfromMidnight | Elapsed time in sec since midnight |
| Event | Mode of event flag (see below for key) |
| BadData | Bad data flag (4 = all good, 2 = ¾ good, 0 = ½ good, -2 = ¾ bad, -4 = all bad) |
| AvgEDA | Average EDA level |
| dAvgEDA | Difference between adjacent windows of Col F |
| RMSEDA | RMS of the EDA signal (filtered to 0.04-0.4 Hz band) |
| dRMSEDA | Difference between adjacent windows of Col H |
| nPksHighThresh | Number of peaks using a high threshold |
| dnPksHighThresh | Difference between adjacent windows of Col J |
| AvgPkHeighthighthresh | Average peak height of peaks found in Col J |
| dAvgPkHeighthighthresh | Difference between adjacent windows of Col L |
| nPksLowThresh | Number of peaks using a low threshold |
| dnPksLowThresh | Difference between adjacent windows of Col N |
| AvgPkHeightlowthresh | Average peak height of peaks found in Col N |
| dAvgPkHeightlowthresh | Difference between adjacent windows of Col P |
| AvgTemp | Average temperature |
| dAvgTemp | Difference between adjacent windows of Col R |
| Activity | Average activity count (uses all 3 axes to derive measurement) |
| dActivity | Difference between adjacent windows of Col T |
| SlopeEDA20s | Slope of avg EDA level (20s window) |
| SlopeEDA120s | Slope of avg EDA level (2min window) |
| NormAvgEDA | Col F / average EDA level from 20:20-0:20 previous |
| ZAvgEDA | Z-score Col F (population is average EDA level from 20:20-0:20 previous) |
| NormRMSEDA | Col H / RMS of EDA signal from 20:20-0:20 previous |
| ZRMSEDA | Z-score of Col H (population is RMS EDA from 20:20-0:20 previous) |
| dispatch | Number of consecutive windows the officer has been dispatched |
| enroute | Number of consecutive windows the officer has been en routeCol |
| arrived | Number of consecutive windows the officer has been at the sceneCol |
| transport | Number of consecutive windows the officer has been conducting a transport |
| idle | Number of consecutive windows the officer has been idle (cleared/cancelled a call) |
| dispatch2 | Number of consecutive windows the officer has been dispatched (2nd or more time within the same call) |
| enroute2 | Number of consecutive windows the officer has been en route (2nd or more time within the same call) |
| arrived2 | Number of consecutive windows the officer has been at the scene (2nd or more time within the same call) |
| transport2 | Number of consecutive windows the officer has been conducting a transport (2nd or more time within the same call) |
| Device | ID of the device used by the officer |

Combined Data Analysis

| | |
|---|---|
| ShiftStartDate | Starting date of the current shift (NOT the current date of the row; if the timestamp goes past midnight on the same day, this will be one day behind the current row) |
| ParticipantID | ParticipantID |
| Shift | Categorical variable identifying officer shifts in chronological order |
| dispatch01 | Dummy coded variable indicating rows during which a dispatch phase occurred |
| en_route01 | Dummy coded variable indicating rows during which an en route phase occurred |
| arrived01 | Dummy coded variable indicating rows during which an officer was on the scene |
| transport01 | Dummy coded variable indicating rows during which a transport phase occurred |
| idle01 | Dummy coded variable indicating rows during which an idle phase occurred |

Multimedia Appendix 2. Combined data for analysis inventory illustrating the combination of variables that result from signal processing and data fusion to prepare for statistical analysis for the Biometrics & Policing Demonstration pilot project.
