# Supplementary material for: Biometrics and Policing: A Protocol for Multichannel Sensor Data Collection and Exploratory Analysis of Contextualized Psychophysiological Response During Law Enforcement Operations
Source: JMIR Res Protoc. 2017 Mar 17;6(3):e44. doi: 10.2196/resprot.7499 (PMC5375974; doi:10.2196/resprot.7499)
Supplement: Multimedia Appendix 4 [file resprot_v6i3e44_app4.pdf]

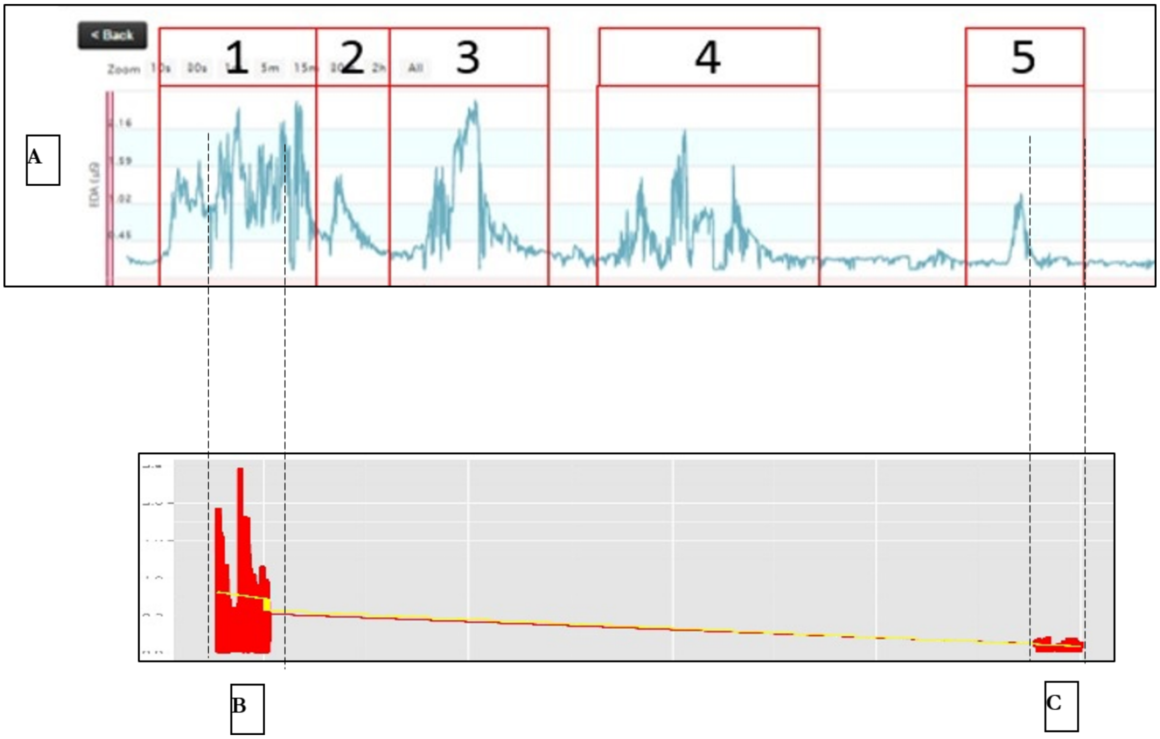

Multimedia Appendix 4. Image showing the stream of electrodermal activity (EDA) data from one officer's shift. Officer's EDA during (A) a full shift (with 5 calls demarcated) and (B) a domestic dispute call vs (C) stolen vehicle recovery. Mixed-model trajectory analysis results suggest statistical model of the difference in EDA between a domestic dispute (B) and a less-stressful call (C). The respective mean EDA levels were 0.77 (SD 0.362) vs 0.11 (SD 0.095), reaching a P<.0001 of statistical significance.
